# Balancing activity, stability and conductivity of nanoporous core-shell iridium/iridium oxide oxygen evolution catalysts

**DOI:** 10.1038/s41467-017-01734-7

**Published:** 2017-11-13

**Authors:** Yong-Tae Kim, Pietro Papa Lopes, Shin-Ae Park, A-Yeong Lee, Jinkyu Lim, Hyunjoo Lee, Seoin Back, Yousung Jung, Nemanja Danilovic, Vojislav Stamenkovic, Jonah Erlebacher, Joshua Snyder, Nenad M. Markovic

**Affiliations:** 1 Department of Energy System, Pusan National University, Pusan, 46241 Korea; 2 Materials Science Division, Argonne National Laboratory, Lemont, IL 60439 USA; 3 Department of Chemical and Biomolecular Engineering, Korea Advanced Institute of Science and Technology, Daejeon, 34141 Korea; 4 Graduate School of EEWS, Korea Advanced Institute of Science and Technology, Daejeon, 34141 Korea; 5 Department of Materials Science and Engineering, Johns Hopkins University, Baltimore, MD 21218 USA; 6 Department of Chemical and Biological Engineering, Drexel University, Philadelphia, PA 19104 USA

## Abstract

The selection of oxide materials for catalyzing the oxygen evolution reaction in acid-based electrolyzers must be guided by the proper balance between activity, stability and conductivity—a challenging mission of great importance for delivering affordable and environmentally friendly hydrogen. Here we report that the highly conductive nanoporous architecture of an iridium oxide shell on a metallic iridium core, formed through the fast dealloying of osmium from an Ir_25_Os_75_ alloy, exhibits an exceptional balance between oxygen evolution activity and stability as quantified by the activity-stability factor. On the basis of this metric, the nanoporous Ir/IrO_2_ morphology of dealloyed Ir_25_Os_75_ shows a factor of ~30 improvement in activity-stability factor relative to conventional iridium-based oxide materials, and an ~8 times improvement over dealloyed Ir_25_Os_75_ nanoparticles due to optimized stability and conductivity, respectively. We propose that the activity-stability factor is a key “metric” for determining the technological relevance of oxide-based anodic water electrolyzer catalysts.

## Abstract (web summary)

Production of affordable, clean hydrogen relies on efficient oxygen evolution, but improving catalytic performance for the reaction in acidic media is challenging. Here the authors show how tuning the nanoporous morphology of iridium/iridium oxide leads to an improvement in activity/stability, compared with conventional iridium-based oxides.

## Introduction

The successful deployment of alternative energy sources depends critically on the development of materials that can efficiently release electrical energy from chemical bonds by the water-splitting reaction in a polymer electrolyte membrane (PEM) electrolyzers (2H_2_O → H_2_ + O_2_) and, in the reverse process, transform the energy stored in the chemical bonds of oxygen and hydrogen back into electrons and water in fuel cells^1, 2^. For both systems, the range of anode and cathode materials that have been explored is diverse, including both precious and non-precious metals^3–10^. In PEM electrolyzers, the catalyst of choice for hydrogen production is well-dispersed Pt nanoparticles^2–4^. However, the selection of materials for the oxygen evolution reaction (OER) is more challenging, a fact that has motivated significant research efforts to develop active anode oxide materials^11–15^. Thus far, the design of active catalysts has been guided by so-called energetic factors that form the backbone of the well-known volcano relationship^6, 7, 9, 16–23^. Despite this well-established energetic design framework, there are clear indications that the design principles should be broadened beyond activity to include both stability^11–13, 24–30^ and conductivity of the oxide materials^27^. Overall, the selection, synthesis and optimization of oxide materials for the OER must centre on the identification and exploitation of property-function correlations and balance activity (e.g., binding energy between the substrate and intermediates^11, 12, 27^), stability (e.g., the rate of dissolution of surface atoms) and conductivity (e.g., the rate of electron transport through oxide materials)—a challenging mission that if overcome may open new avenues for the efficient and cost-effective production of hydrogen and oxygen through electrolytic processes.

Among the many synthesis methods that have been developed to optimize the efficiency of the OER, the most prominent is the electrochemical etching of multimetallic alloys and the formation of various porous architectures, with Ir-based oxides identified as particularly promising for technological applications^12, 15, 31–35^. Selective etching or dealloying is traditionally defined as the electrochemical dissolution of the less noble (LN) component(s) and concomitant surface diffusion and island formation of the more-noble (MN) component^31, 36^. Porosity begins to evolve as the LN-rich alloy separates from the formed MN islands, with layer-by-layer etching of the LN component limited by the rate of MN surface diffusion, moving the etch front into the bulk of the material^35^. Current fundamental understanding of the dealloying process has facilitated the development of a broad range of catalytic materials, with deliberate manipulation of dealloying conditions promoting the strict control of material activity^33, 36–38^. However, it is also clearly of importance to explore the possible role of pore architecture on the stability of atoms within the remaining structure, as well as on the conductivity of such oxide materials.

Here we report the design of Ir-based nanoporous architectures, formed through the dealloying of a series of Ir_*x*_Os_(1−*x*)_ alloys, that exhibit an exceptional balance between the OER activity and stability, as quantified by a metric introduced here, the Activity-Stability Factor (ASF), expressed as the ratio between activity for OER and stability of the oxide material. We first demonstrate that the morphology of Ir_*x*_Os_(1−*x*)_ dealloyed thin films (dtf-Ir_*x*_Os_(1−*x*)_) is a consequence of the fast dealloying of Os and the concomitant slow dissolution of Ir. The dealloyed Ir_25_Os_75_ catalyst exhibits a factor of ~30 improvement in the ASF relative to state-of-the-art Ir-based oxide materials, as a consequence of its nanoporous Ir-oxide/Ir-metal morphology rather than by Os-induced electronic effects on Ir surface atoms. Furthermore, the impact of Ir-oxide conductivity on the ASF is resolved by comparing the OER electrochemistry of dealloyed thin films and dealloyed/agglomerated nanoparticles. Although possessing a higher surface-area-to-volume ratio relative to dtf-Ir_25_Os_75_, nanoparticles demonstrate a lower intrinsic activity due to their lower conductivity, which arises from the high fraction of oxide-oxide interfaces that form between nanoparticles. We conclude that the ASF is a key metric for determining the technological relevance of oxide-based anodic water electrolyzer catalysts.

## Results

### Synthesis of nanoporous iridium and mechanism of IrOs dealloying

**Fig. 1:**
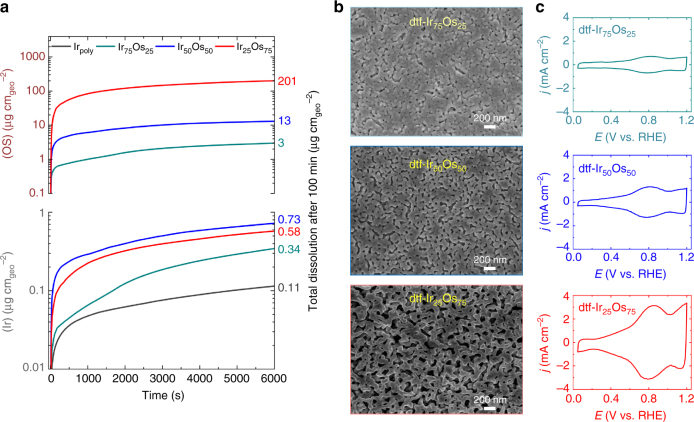
In situ dealloying dynamics of Ir_*x*_Os_(1−*x*)_ alloys. **a** Comparison between total Os (top curves) and Ir (bottom curves) dissolution measured in situ with SPRDE-ICP-MS for Ir_75_Os_25_ (green), Ir_50_Os_50_ (blue) and Ir_25_Os_75_ (red) and Ir-poly (black) under the same conditions (1 mA cm^−2^ in 0.1 M HClO_4_ at 25 °C) demonstrating significant dissolution of Os, accompanied by a small Ir loss during the dealloying protocol. **b** Representative SEM images of dealloyed Ir_*x*_Os_(1−*x*)_ catalysts revealing a porous architecture, with varying pore size (10–100 nm) and surface-to-volume ratio generated through the dealloying process. **c** Corresponding cyclic voltammograms (CVs) of dealloyed materials (0.1 M HClO_4_ at 50 mV s^−1^), showing the same pseudocapacitive (OH_ad_) profiles and peak position but with increasing current density for higher Os contents in the alloy, indicative of higher ECSA and negligible electronic effect of Os content in the alloy

We begin by utilizing an in situ Stationary Probe Rotating Disk Electrode (SPRDE) coupled to an Inductively Coupled Plasma-Mass Spectrometer (ICP-MS) as a unique method for monitoring the kinetics of dealloying processes in the alloy containing both LN and MN components^29^. The rates of dissolution and the corresponding change in morphology during the dealloying of several thin films of Ir_*x*_Os_(1−*x*)_ (hereafter denoted as dtf-Ir_*x*_Os_(1−*x*)_) with different compositions (*x* = 9 to 75 at%) are explored in acidic media, as fully described in the Methods section. As summarized for three particular compositions in Fig. 1a, Supplementary Fig. 1 and Supplementary Note 1, SPRDE–ICP–MS data reveals several important, time-dependent, dissolution profiles of Os and Ir for the Ir_*x*_Os_(1−*x*)_ thin films. In particular, the rate of Os dissolution during dealloying is exceptionally fast in the first 1000 s. (top part of Fig. 1a), with the total amount of dissolved Os increasing in the order dtf-Ir_75_Os_25_ (~3 µg cm^−2^) < dtf-Ir_50_Os_50_ (~13 µg cm^−2^) < dtf-Ir_25_Os_75_ (~200 µg cm^−2^). The high rate of Os dissolution at low pH values (Supplementary Fig. 1) is a consequence of the inability of Os to form a stable oxide at potentials above 1.0 V, behavior that is reminiscent of that observed for bulk Os electrodes^11, 39^. Markedly, slower rates of Ir dissolution were observed (bottom part of Fig. 1a), with the amount of dissolved Ir increasing in the order Ir-poly (~0.11 µgcm^−2^) < dtf-Ir_75_Os_25_ (~0.34 µg cm^−2^) < dtf-Ir_25_Os_75_ (~0.58 µg cm^−2^) < dtf-Ir_50_Os_50_ (~0.73 µg cm^−2^). Note that the maximum Ir dissolution during dealloying corresponds to the alloy with the highest number of Ir-Os bonds (Ir_50_Os_50_). Importantly, all dtf-Ir_*x*_Os_(1−*x*)_ films exhibit a higher Ir dissolution rate relative to Ir-poly (Fig. 1a), in contrast to other dealloying systems where dealloying conditions lie within the region of passivity for the MN component^33, 38^. It is conceivable that, besides intrinsic thermodynamic and kinetic arguments^12^, one key reason to account for this unexpected Ir instability is the concomitantly high rates of Os dissolution. As we shall see in the subsequent section, a substantial difference in the dissolution rates between Os (LN) and Ir (MN) elements and small, yet continuous, dissolution of the MN component during dealloying leads to the formation of an unexpectedly large porous morphology of the remaining Ir skeleton.

In general, the porosity length scale for nanoporous metals evolved during dealloying is dependent upon the rate of surface diffusion of the MN component, defined by the dealloying conditions and the intrinsic bonding properties of the metal^34^. As is observed for Pt alloy systems^32, 38^, the pore size obtained through dealloying of Ir-based alloys is expected to be below 10 nm based on the low rate of Ir surface diffusion anticipated from its high melting point. Surprisingly, scanning electron microscopy (SEM) images depicted in Fig. 1b, Supplementary Fig. 2 and Supplementary Note 2 show that the pores formed are considerably larger – on the order of 100 nm in diameter for Ir_25_Os_75_. As mentioned earlier, the noticeably larger pore size in dtf-Ir_*x*_Os_(1−*x*)_ likely derives from a modification of the proposed dealloying mechanism due to the large disparity in the rates of Os and Ir dissolution. Additional Ir instability is induced by the high rates of Os dissolution, which leads to the development of larger pits than should otherwise be possible. It should be emphasized that the proposed dealloying mechanism is only possible based on the ability to measure such small rates of Ir dissolution. Nevertheless, the consequence from this dealloying is the formation of a nanoporous morphology with exceptionally high electrochemically active surface area (ECSA). By analogy with other systems, ECSA values are evaluated from cyclic voltammograms (CVs) shown in Fig. 1c, Supplementary Fig. 3 and Supplementary Note 3. We note that the ECSA values determined by the above method can give only relative changes in surface area as compared to a “flat” surface; however, these relative changes are what matter when benchmarking performance against established catalyst materials, and provide a fair comparison between the different materials investigated in this study. Thus, from Table 1, the ~ 50-fold increase in ECSA observed for dtf-Ir_25_Os_75_ relative to Ir-poly is consistent with the presence of a bicontinuous porosity and, thus, an increase in the overall density of Ir surface sites.

**Table 1:** Catalytic properties of relevant metal oxide materials for OER

Material	ECSA/cm^2^	Activity^a^(J)/mA cm_geo_^−2^	Specific Activity^a^/mA cm^−2^	Dissolution^a^(S)/ppb h^−1^	Activity-Stability Factor (×10^3^)^a^
Ir_-poly_	0.91	0.04	0.013	0.57	0.71
dtf-Ir_25_Os_75_	45.66	2.10	0.013	1.03	20.67
dnp-Ir_50_Os_50_	52.51	3.34	0.018	1.82	18.60
dtf-Ir_50_Ru_50_	1.84	0.32	0.049	2.96	1.09
Ru_-poly_	3.16	3.23	0.289	178.0^b^	0.18
dtf-Ru_25_Os_75_	46.54	18.75	0.114	399.9^b^	0.48

^a^Obtained at a constant overpotential of 0.25 V

^b^Determined for Ru dissolution

Importantly, post-dealloying ICP-MS and secondary ion mass spectrometry (SIMS) analysis of dtf- Ir_*x*_Os_(1−*x*)_ samples reveals that the dealloyed material in the near-surface region (within a 15–20 nm thickness) has a residual Os content of <0.05 at.% (Supplementary Fig. 4 and Supplementary Note 4), suggesting that the influence of the remaining Os on the electronic properties of the surface Ir should be negligible and that the surface adsorption properties should be similar to Ir-poly. This supposition is supported by the fact that the position of the pseudocapacitive peak at 0.8 V, which is associated with the formation of adsorbed hydroxide (OH_ad_), is similar to that of Ir-poly regardless of the initial alloy composition (Fig. 1c). An important consequence of this conclusion is that the intrinsic rate (specific current density) of the OER on dealloyed dtf-Ir_*x*_Os_(1−*x*)_ surfaces should be the same as that observed for Ir-poly. As a result, any “improvement” in the OER should be related to ECSA, rather than the electronic effects that are frequently used to justify the improved activity of bimetallic systems^8^. However, the beneficial increase in the number of active surface sites provided by higher ECSA is completely nullified if the amount of dissolved Ir scales linearly with surface area; an issue which has been completely overlooked for electrochemical systems due to a lack of appropriate metrics that can account for both activity and stability. Therefore, in the following, we develop a method of quantifying activity-stability relationships and use this metric to understand the effects of morphology on the intrinsic performance of OER catalyst materials.

### Activity-stability relationships for OER on dtf-Ir_*x*_Os_(1−*x*)_

The OER is a rather complex process involving the formation of many reaction intermediates before H_2_O is completely converted to O_2_/H^+^. Thus far, many descriptors have been used to express the overall catalytic activities of the OER, including the oxygen adsorption energy in gas phase environments, the catalyst-OH bond strength^19^, the number of *e*_*g*_ electrons^23^, the lattice spacing in metal electrodes and the covalence of oxygen-metal bonds^40^. Although these studies have made a significant impact in rationalizing which surface properties may govern the variations in reactivity from one catalyst to the next, fundamental understanding of the elementary processes involved in the catalytic transformation of water to di-oxygen is still lacking. This is because catalysts are not stable during the OER, and the nature and density of active sites may also vary during the reaction. As a consequence, a rigorous kinetic analysis of the OER lies outside the scope of the present discussion. As correlations between the activity and electronic properties of monovalent oxides during the OER have been addressed extensively in our prior work^11^, we will instead focus on quantifying the relationship between the activity and stability of dealloying-induced nanoporous oxide architectures and how this relationship may aid in the future design of anode materials for acid-based electrolyzers.

**Fig. 2:**
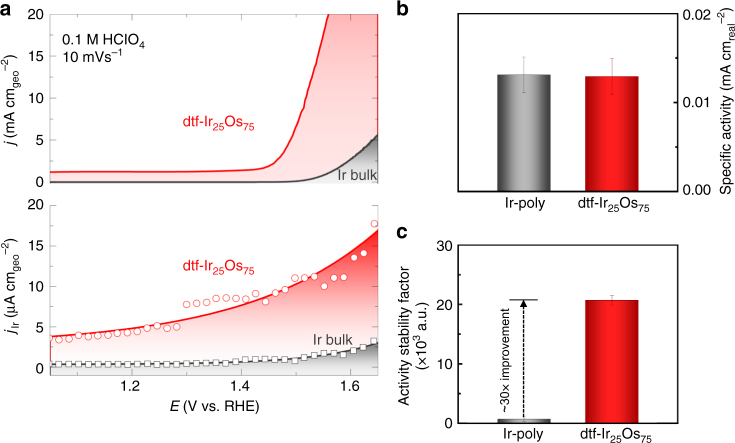
Activity-stability relationships for dtf-IrxOs_(1−*x*)_ catalysts. **a** OER activity (top graph) and in situ monitoring of simultaneous Ir dissolution (bottom graph) in acid media revealing that the OER is always accompanied by dissolution of the oxide material (Ir-poly in black; dtf-Ir_25_Os_75_ in red). **b** Specific activity obtained after proper normalization by ECSA showing that both catalysts have the same activity, suggesting that morphological, rather than electronic, effects control the OER (Ir-poly in black; dtf-Ir_25_Os_75_ in red). **c** The ratio between activity and stability as quantified by ASF showing the performance of dtf-Ir_25_Os_75_ (red) when compared to Ir-poly (black). Error bars are the standard deviation after 5 experiments

We begin by exploring whether it is possible to “tailor” the nanopore size (e.g., ECSA of the remaining Ir- skeleton) by controlled dealloying of dtf-Ir_*x*_Os_(1−*x*)_ such that the balance between activity and stability is significantly higher than state-of-the-art Ir-poly or other Ir-based and Ru-based alloy systems. In line with our previous report^29^, analysis of in situ SPRDE-ICP-MS data enables the deconvolution of the experimentally measured faradaic currents for the OER, expressed as current per geometric surface area (Fig. 2a), from simultaneous dissolution of Ir, which is expressed as the equivalent “ionic” dissolution current (see bottom part of Fig. 2a and Methods section). We emphasize that in situ determination of corrosion rates gives the true measure of stability in real time, as opposed to traditional stability tests that require several hours of continuous polarization and are highly influenced by catalyst loading on the electrode. From the data presented in Fig. 2, several points are important to highlight. First, the OER is always accompanied by dissolution of the elements comprising the catalyst, a fact that is underestimated in many mechanistic discussions of the OER. As observed for nearly all OER electrocatalysts, the activity of surface atoms is inversely proportional to their stability (Supplementary Fig. 5 and Supplementary Note 5). Second, based on the fact that the Ir dissolution current is ~ 3 orders of magnitude smaller than that for the OER, we conclude that the contribution of Ir corrosion current to the overall measured Faradaic current is negligible (e.g. Faradaic efficiency greater than 99.9%). Third, ECSA normalization of the OER current yields almost the same specific activity for both dtf-Ir_25_Os_75_ and Ir-poly (Fig. 2b, Supplementary Fig. 6, Supplementary Note 6 and Table 1), suggesting that, as in the case of Ru-Ir systems^12^, electronic effects play a negligible role in the overall reaction rates. This also highlights the importance of properly normalizing any OER current by the active surface area before assigning any role to the electronic effects associated with a surface evolved from the dealloying of a multi-component alloy. Significantly, although the ECSA of dtf-Ir_25_Os_75_ is ~ 50 times higher than that of Ir-poly, the amount of Ir dissolution is only 4 times higher than that observed for Ir-poly.

There are two possible explanations for the higher overall stability of the dtf-Ir_25_Os_75_ relative to Ir-poly that is implied by the non-linear relationship between ECSA and Ir dissolution on these two surfaces. First, the small, yet continuous dissolution of Ir from the surface oxide can lead to significant surface faceting, leaving only the most stable Ir-oxide surface orientation behind. That would be in line with the tendency of the remaining surface atoms to rearrange in the energetically most stable (111) like facets^41^. This is also in agreement with the observation that well-ordered (111) surfaces of Ir and Ru are much more stable than the corresponding “rough” polycrystalline electrodes^11^. Second, it is also plausible that the concentration gradient of Ir ions inside the nanopores of the dtf-Ir_25_Os_75_ is substantially lower than for the “flat” Ir-poly electrodes, resulting in a lower thermodynamic driving force for Ir dissolution within the nanoporous structures (as has been previously observed on “cracked” Ru materials^42^). Admittedly, a true increase in the stability of nanoporous films has still not been fully resolved and it will require many more systematic experimental and modeling studies. Regardless, the data presented in Fig. 2 unambiguously show that the balance between activity and stability can be addressed through the manipulation of the material morphology, which has rarely been discussed in the existing literature. Below, we quantify such activity-stability relationships and, in doing so, rationalize our claim that future catalyst design for the OER cannot be judged solely based on the activity (current density at given overpotential) of anode oxide materials.

As a starting point, we propose that the proper and correct account of activity and stability for a given material must be defined as the ratio between the rate of di-oxygen production (expressed as current density *J*) and the rate of metal dissolution during OER (equivalent dissolution current density, *S*). This activity-stability metric will hereafter be denoted as the Activity-Stability Factor, ASF (Eq. 1),

1 $${\mathrm{ASF}} = \left. {\frac{{J - S}}{S}} \right|_{\mathrm{\eta }},$$

where at constant overpotential (η), the best materials for the OER should be those with higher ASF values. Although it may appear that by following the Faradaic efficiency of OER can provide insights into the ratio between activity and stability^43^, the fact that oxygen can be evolved from both water and the oxide lattice^15, 44^ limits the accuracy of any quantitative assessment of the ratio of OER activity and stability of oxide materials. Figure 2c and Table 1 contain representative values of the ASF for the range of bulk and thin-film materials explored under the same experimental conditions (c.a. *η* = 250 mV). Two trends are clearly evident in Table 1. First, the ASF is almost 4 times higher for Ir-poly than for Ru-poly (Table 1), as the higher “activity” of Ru is offset by its significantly lower stability^11, 12, 42^. To the best of our knowledge, the ASF provides a true quantitative validation of the accepted empirical conclusion that Ru-based materials are not technologically relevant for efficient oxygen production in water electrolyzers. Second, marked differences between Ir-poly, dtf-Ir_*x*_Os_(1−*x*)_ and other Ru-based materials (used here for comparison) are observed; with ASF values that increase in the order: Ru-poly < dtf-Ru_50_Os_50_ < Ir-poly << dtf-Ir_25_Os_75_. Note that dtf-Ir_25_Os_75_ shows a 30-fold increase in the ASF over Ir-poly (Fig. 2c), 20-fold over dtfIr_50_Ru_50_ and 35-fold over dealloyed dtf-Ru_25_Os_75_, the latter having a similar ECSA (from Table 1 ~ 45 cm^−2^) but slightly different pore size and morphology (Supplementary Fig. 7 and Supplementary Note 7). We note in passing that the ASF for two other dtf-Ir_*x*_Os_(1−*x*)_ (results not shown) is three times lower than that found for dtf-Ir_25_Os_75_, confirming that dtf-Ir_25_Os_75_ possesses an ideal balance between activity and stability. Taken together, these observations clearly demonstrate that the ASF is a concise, accurate metric that can aid the assessment and design of technologically relevant electrocatalysts with potential applicability beyond anodic OER oxide materials. Given the clear benefits of nanoporosity and high ECSA to the ASF of dtf-Ir_25_Os_75_, the question arises whether further improvement of the ASF for the OER can be achieved by employing the corresponding high surface area IrO_*x*_ nanoparticles, that possess a morphology that more closely resembles that of the sintered agglomeration of oxide particles that are commonly used in water electrolyzer systems^45, 46^.

### Morphology controlled activity-conductivity relationships

Traditional OER oxide anode electrodes are composed of a sintered agglomeration of nanoparticles mixed with some ionomer, such as Nafion, as carbon materials are unstable at OER operation potentials^45, 46^. To draw direct comparisons between the nanoporous dtf-Ir_25_Os_75_ and sintered electrodes, we have synthesized Ir_*x*_Os_(1−*x*)_ nanoparticles through a colloidal approach with varied composition (hereafter denoted as dnp-Ir_*x*_Os_(1−*x*)_). Provided that the best results are obtained for the dnp-Ir_50_Os_50_, rather than dnp-Ir_25_Os_75_, we restrict the discussion here to a brief summary of the results for the OER on the former in acid electrolyte.

**Fig. 3:**
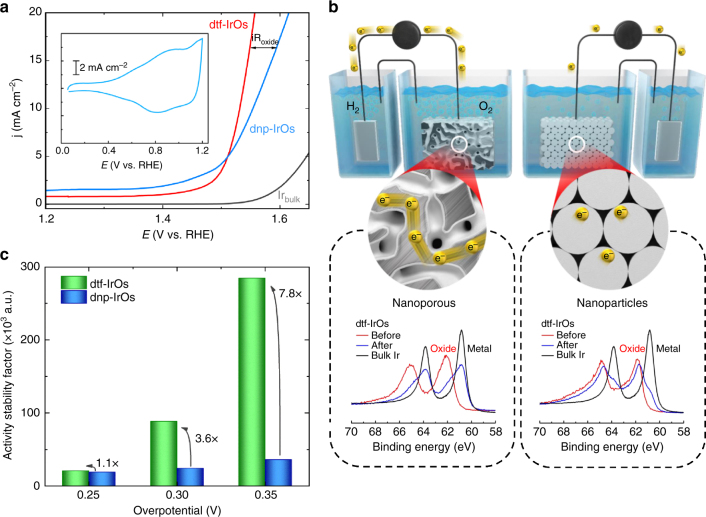
Activity-conductivity relationships in dealloyed thin-film and nanoparticles. **a** Comparison between OER polarization curves for Ir-poly, dtf-Ir_25_-Os_75_ and dnp-Ir_50_Os_50_, indicating that conductivity limitations are observed for dnp-Ir_50_Os_50_ at higher current densities (denoted as iR_oxide_). Inset shows the corresponding CV with a similar OH_ad_ profile as in Fig. 1c. **b** XPS sputter etching experiments demonstrating that the dtf-Ir_25_Os_75_ consists of an IrO_*x*_ shell with Ir-metallic core, in contrast to dnp-Ir_50_Os_50_ that consists entirely of IrO_*x*_. Schematic illustrates the impact of multiple oxide-oxide interfaces (present on dnp-Ir_50_Os_50_ electrodes) on conductivity. **c** Change in Activity-Stability Factor (ASF) values with overpotential for dtf-Ir_25_Os_75_ and dnp-Ir_50_Os_50_ highlighting the importance of balancing activity-stability-conductivity properties of oxide materials for the OER

A representative TEM image, depicted in the Supplementary Fig. 8 and Supplementary Note 8, reveals that the as-synthesized Ir_50_Os_50_ nanoparticles are 2–3 nm in diameter with a narrow size distribution. These nanoparticles are then dealloyed using the same protocol described for dtf-Ir_*x*_Os_(1−*x*)_ alloy systems. Although porosity may not evolve in the same way as that observed for thin films due the small particle diameter^32, 38^, it is reasonable to anticipate that the dealloying of nanoparticles is also governed by the fast dissolution of Os. Indeed, CVs of dnp-Ir_50_Os_50_ and dtf-Ir_25_Os_75_ have a similar shape with the same position of the OH_ad_ peaks at ~ 0.8 V (Supplementary Fig. 9 and Supplementary Note 9). Further inspection of the CVs reveals that the pseudocapacitive charge for the dnp-Ir_50_Os_50_ is higher than that for dtf-Ir_25_Os_75_, which can be accounted for by a higher ECSA for the agglomerated nanoparticle electrode (see Fig. 3a and Table 1). If the electronic properties of Ir are the same for both systems, which is a reasonable assumption based on the position of the OH_ad_ peaks, ECSA normalization should yield similar activities for both nanoparticles and dealloyed thin films. Surprisingly, Fig. 3a shows that the OER activity is higher for dtf-Ir_25_Os_75_ than that for dnp-Ir_50_Os_50_ above *η* = 280 mV. This result suggests that the ECSA itself may not be the only factor influencing the measured OER current. One possible reason for the lower observed specific activity is a difference in conductivity between dtf-Ir_25_Os_75_ and dnp-Ir_25_Os_75._ The current-voltage curve for dnp-Ir_50_Os_50_ provides some evidence that this may be the case, as it does not exhibit the characteristic exponential behavior that is expected for highly conductive materials^27, 47^, especially at high current densities. Below we investigate the conductivity, carrier mobility and surface chemistry of the dnp-Ir_50_Os_50_ and dtf-Ir_25_Os_75_ electrodes in order to identify the origin of the observed differences in activity.

It is well established that both the carrier mobility and carrier density are the main factors contributing to the overall electrode conductivity. The values of these two parameters for oxidized dnp-Ir_50_Os_50_ and dtf-Ir_25_Os_75_ specimens are established by using 4-probe van der Pauw measurements, and the results are summarized in Supplementary Table 1. Although both materials exhibit the same carrier density (~ 1.2 × 10^19^ cm^−3^), the carrier mobility of dtf-Ir_25_Os_75_ (8.99 × 10^−2^ cm^2^ V^−1^ s^−1^) is significantly higher than that of dnp-Ir_50_Os_50_ (1.11 × 10^−2^ cm^2^ V^−1^ s^−1^). This suggests that the lower conductivity of dnp-Ir_50_Os_50_ aggregates (3.17 × 10^−2^ S cm^−1^) relative to that of dtf-Ir_25_Os_75_ (1.6 × 10^−1^ S cm^−1^) is governed by the carrier mobility. Importantly, XPS sputter etching experiments (shown in Fig. 3b) indicate that the dtf-Ir_25_Os_75_ structure is “core-shell” in nature, with a metallic Ir core and an IrO_*x*_ shell with a thickness of a few nanometers (see also the schematics in Fig. 3b). In contrast, the XPS sputter etching profile of the agglomerated dnp-Ir_50_Os_50_ electrode continued to show an IrO_*x*_ species throughout the entire etching depth (see schematics in Fig. 3b). This is likely due to the small diameter of the nanoparticles (2–3 nm), which makes them susceptible to almost full conversion to the high valent oxide at OER operational conditions. We therefore propose that the difference in specific activity between the two structures arises from the fact that dtf-Ir_25_Os_75_ contains a three-dimensionally interconnected, bicontinuous, metallic-core structure, which provides a low resistance electron transport channel below the few nanometers of resistive oxide “shell”. This is in contrast to the highly oxidized core and surface of the agglomerated dnp-Ir_50_Os_50_ electrodes which forms a high resistance electron conduction pathway across many oxide-oxide interfaces, resulting in the lower observed catalytic activity relative to dtf-Ir_25_Os_75_.

Turning now to comparing the difference in activity-stability factors between dtf-Ir_25_Os_75_ and dnp-Ir_50_Os_50_, Fig. 3c shows selected ASF values at three-electrode overpotentials, again obtained under the same experimental conditions. The difference in ASF becomes more visible at higher overpotentials; e.g., while the performance of dtf-Ir_25_Os_75_ is only 1.1 times better than dnp-Ir_50_Os_50_ at *η* = 0.25 V, the ASF is 3.6 and 7.8 times higher at *η* = 0.3 V and *η* = 0.35 V, respectively. The observed overpotential-dependence can be anticipated as the role of the electrode conductivity, which is more significant at higher current densities, both through-plane and in-plane. In view of these results, it is clear that the efficiency of oxygen production in electrolyzers is also governed by the intrinsic conductivity of oxide materials. Even more importantly, the selection of anode oxide materials for efficient and sustained oxygen production in water-based electrolyzers should be based on the maximization of the ASF values discussed above for a given conductivity.

## Discussion

We find that the highly conductive, nanoporous architecture of an Ir oxide shell on a metallic Ir core, formed through the fast dealloying of Os from an Ir_25_Os_75_ alloy shows a factor of ~ 30 improvement in the ASF relative to conventional Ir-based oxide materials and an 8-fold improvement relative to dealloyed Ir_50_Os_50_ nanoparticles. Our work shows that the ASF may serve as a universal metric that encompasses both the activity and stability of oxide materials for the OER. Therefore, we propose that the selection of anode oxide materials for water electrolyzers should be based on ASF values rather than the conventionally used measurements of activity, expressed either as specific or mass activity. The importance of ASF goes well beyond the OER electrocatalysis, as it can serve as a fundamental “performance metric” for selecting the best materials for various types of electrochemical processes employed in renewable energy technologies.

## Methods

### Synthesis of polycrystalline bulk metal

Polycrystalline bulk metal (Ir, Ru, Ir_25_Ru_75_, and Ir_25_Os_75_) surfaces were prepared by the vacuum plasma arc-melting technique (ACI alloy, 6 mm diameter, 6 mm thickness, 99.99% purity) and polished down to 0.05 µm with alumina powder (Buehler powders and grinding paper) until the formation of a mirror-like surface. All the bulk metals were annealed in a RF furnace (Ambrell, Easyheat 0224) in Ar-3%H_2_ for 10 min at 1100 °C before testing.

### Synthesis of thin film and nanoparticle catalysts

Thin-film surfaces were prepared by using the solution deposition technique on a polycrystalline Ir pellet (ACI alloy, 6 mm diameter, 6 mm thickness, 99.99% purity). Iridium chloride hydrate (Alfa aesar, 99.9%) 0.03 μmol, osmium chloride hydrate (Alfa aesar, 99.99%) 0.09 μmol and ruthenium chloride hydrate (Sigma-Aldrich, 99.98%) 0.09 μmol were employed as the metal sources. The final metal deposition process was carried out in the RF furnace in Ar-3%H_2_ for 70 s at 1100 °C. Nanocrystals of IrOs alloy were prepared using metal carbonyl precursors. 52 mg of dodecacarbonyltriosmium (Os_3_(CO)_12_, Alfa aesar, 99%) and 47.6 mg of dodecacarbonyltetrairidium (Ir_4_(CO)_12_, Alfa aesar, 98%), and 76.7 μL trioctylphosphine (Sigma-Aldrich, 97%) were introduced into 10 mL oleylamine (Sigma-Aldrich, 70%) in a three neck round bottom flask. The solution was homogenized by stirring for 1 h at 80 °C. Then, the temperature was elevated to 220 °C with N_2_ flow. After stirring for an additional 2 h, the reaction was quenched by cooling abruptly to room temperature. The resulting nanoparticles were washed with n-hexane and ethyl alcohol.

### XRD

The crystalline structures of the prepared samples were determined with an X-ray diffractometer (XRD, Bruker, D8 advance) with CuKα target (*λ* = 1.54056) in 2*θ* = 10–90° (0.004° s^−1^) at room temperature. XRD spectra for Ru-poly, Ir-poly, and IrOs alloys are shown in Supplementary Fig. 10 and Supplementary Note 10.

### SEM and TEM

The morphologies of the polycrystalline/thin film and nanoparticle samples were observed with field emission scanning electron microscopy (FE-SEM, Zeiss, SUPRA25) using a 10 kV accelerating voltage and aberration-corrected transmission electron microscopy (cs-corrected TEM, FEI, Titan cubed G2 60–300) with a 300 kV accelerating voltage, respectively.

### XPS sputter etching depth profiling

The electronic energy state was measured by depth profiling samples with X-ray photoelectron spectroscopy (XPS) accompanied by ion-beam sputtering by using a synchrotron beam in the 8A2 undulator beamline in Pohang light source (PLS). The beam flux was 10^11^~10^12^ photons per s and the depth profile was recorded with ion-beam sputtering using 1 keV of Ar^+^.

### Conductivity analysis

The conductivity of thin films or nanoparticles was measured by deposition on a Si wafer, using the same preparation condition for all the tested samples, followed by the 4-probe van der Pauw method on a Hall effect measurement system (Ecopia, HMS-5000/AMP55T) under 1 mA of current and 0.55 T of magnetic field at room temperature.

### Electrochemical experiments

All the electrochemical tests and dealloying processes were performed with a potentiostat (Biologic, VSP) and a standard three-electrode electrochemical cell with a rotating disk electrode (RDE) setup and the prepared pellet-type sample as the working electrode, a Pt wire as the counter electrode, and Ag/AgCl for reference electrode in nitrogen-saturated 0.1 M HClO_4_ solution. The electrode potential is reported against the reversible hydrogen electrode (RHE), calibrated from a separate experiment. The potential sweep ranges and rates were 0.05–1.2 V (vs RHE), 50 mV s^−1^ for cyclic voltammetry (CV), 0.05–1.8 V (vs RHE) and 10 mV s^−1^, with a rotation speed of 1600 r.p.m. for OER measurements. Stability of catalysts were also evaluated by steady state polarization curves obtained over extend periods of time at ~ 1 mA cm^−2^ (Supplementary Fig. 11 and Supplementary Note 11). All the polarization curves were obtained with solution iR compensation. The electrochemical dealloying process was carried out with the application of constant current (1 mA cm^−2^) for 100 min (6000 s). All the tests were conducted at room temperature and ambient pressure.

### In Situ ICP-MS experiments

Simultaneous measurement of the activity and stability was done by the Stationary Probe Rotating Disk Electrode method, by analyzing the solution directly into an ICP-MS (Perkin Elmer, NexION 300D)^29^. Ir and Os were simultaneously measured in the ICP-MS while the working electrode was controlled by the potentiostat. This allowed monitoring of the dissolution during the dealloying process as well as during the OER, with the same methodology as already described. For high turnover dissolution testing, ex situ ICP-MS measurements were also conducted (ELAN, UP-213), confirming the in situ results.

### Data availability

The data that supports the findings of this study are available upon request from the corresponding authors.

## Electronic supplementary material

Supplementary Information
