# Supplementary material for: Balancing activity, stability and conductivity of nanoporous core-shell iridium/iridium oxide oxygen evolution catalysts
Source: Nat Commun. 2017 Nov 13;8:1449. doi: 10.1038/s41467-017-01734-7 (PMC5682288; doi:10.1038/s41467-017-01734-7)
Supplement: Supplementary file 1 — Supplementary Information [file 41467_2017_1734_MOESM1_ESM.pdf]

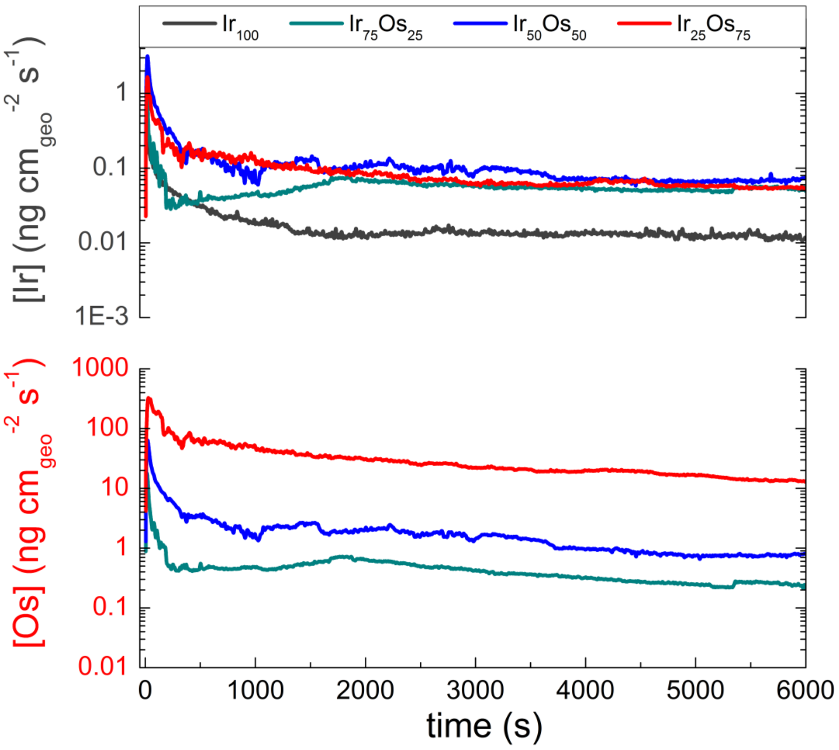

Supplementary Figure 1: De-alloying dynamics showing continuous dissolution of Ir and Os while the electrode is polarized to provide 1mA cm^{-2} during the full 6000s. Electrolyte 0.1M $HClO_4$ and 25ºC.

Supplementary Note 1

De-alloying dynamics: After immersion, real-time dissolution rates of Ir and Os during de-alloying procedure is depicted in **Supplementary Figure 1**. As expected from the nature of the process, continuous dissolution is observed immediately after polarization begins. The highest dissolution rates, given by the ion concentration in solution stream that enters the ICP-MS times its flow rate, are observed right at the beginning of polarization, monotonically decreasing with de-alloying time. For all IrOs alloy contents, this reflects the initial depletion of Os from the outermost surface layers and consequent passivation of Ir over Os, defining the material morphology evolution later observed as a unique porous structure.

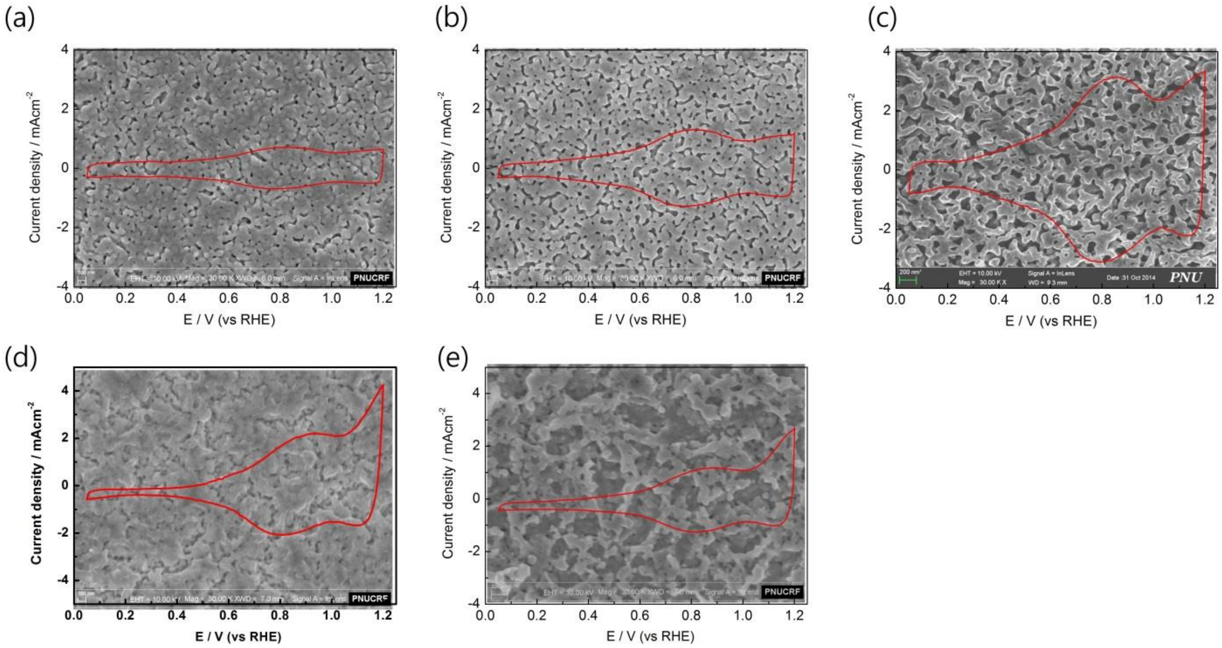

Supplementary Figure 2: Scanning electron micrograph (SEM) and cyclic voltammetry (CV) of the dealloyed nanoporous (a) dtf-$Ir_{75}Os_{25}$, (b) dtf-$Ir_{50}Os_{50}$, (c) dtf-$Ir_{25}Os_{75}$, (d) dtf-$Ir_{17}Os_{83}$, and (e) dtf-Ir_9Os_{91}. Images are 4 x 3 μm^2.

Supplementary Note 2

Morphology variation with alloy composition: The nanoporous structures were developed by the dealloying process and were varied with the alloy composition. It is obvious that a lower content of Os leads to an insufficiently developed nanoporous structure as shown in the case of dtf-$Ir_{75}Os_{25}$ and dtf-$Ir_{50}Os_{50}$. On the other hand, an excessive content of Os results in a collapsed and agglomerated structure as shown in the case of dtf-$Ir_{17}Os_{83}$, and dtf-Ir_9Os_{91}. A distinctive, well-ordered nanoporous structure was formed for dtf-$Ir_{25}Os_{75}$. The morphology was directly related to the value of ECSA measured based on the electrochemical double-layer capacitance. The electrochemical double-layer capacitance (measured from CV curves overlaid on SEM images) was in a good agreement with the morphology, indicating that the dtf-$Ir_{25}Os_{75}$ has an optimal composition with respect to nanoporous structure formation.

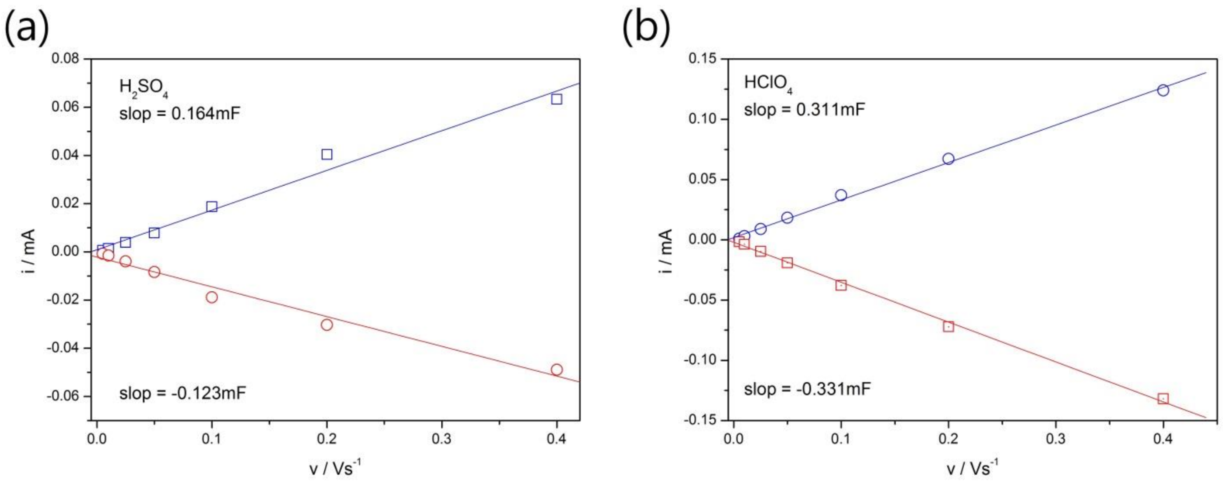

Supplementary Figure 3: Double-layer capacitance measurements to determine the electrochemical surface area (ECSA) for dtf-IrOs catalyst from voltammetry in 0.05 M H_2SO_4 and 0.1M $HClO_4$.

Supplementary Note 3

ECSA measurement: Cyclic voltammograms were measured in a non-Faradaic region of the potential sweep at multiple scan rates: 0.005, 0.01, 0.025, 0.05, 0.1, 0.2, and 0.4 V/s. All current is assumed to be due to capacitive charging. The cathodic (red open circle) and anodic (blue open square) charging currents measured at 0.3 V vs RHE are plotted in Figure S3 as a function of scan rate. The double-layer capacitance of the system is taken as the average of the absolute value of the slope of the linear fits to the data, according to previous literature (**Supplementary Reference** [1]). We note that although these reference capacitance values are important for "true" surface area determination, the ratio between ECSA for different Ir materials is independent of the reference capacitance chosen, i.e. measurements of relative ESCA via this technique enable valid comparisons between materials. Therefore, even though the electrochemical double layer capacitance is not a perfect measurement for accurate quantification the true ESCA, it is certainly better than AFM-based methods and is more than sufficient to facilitate direct comparison of the

electrodes generated in this study when measured under identical conditions.

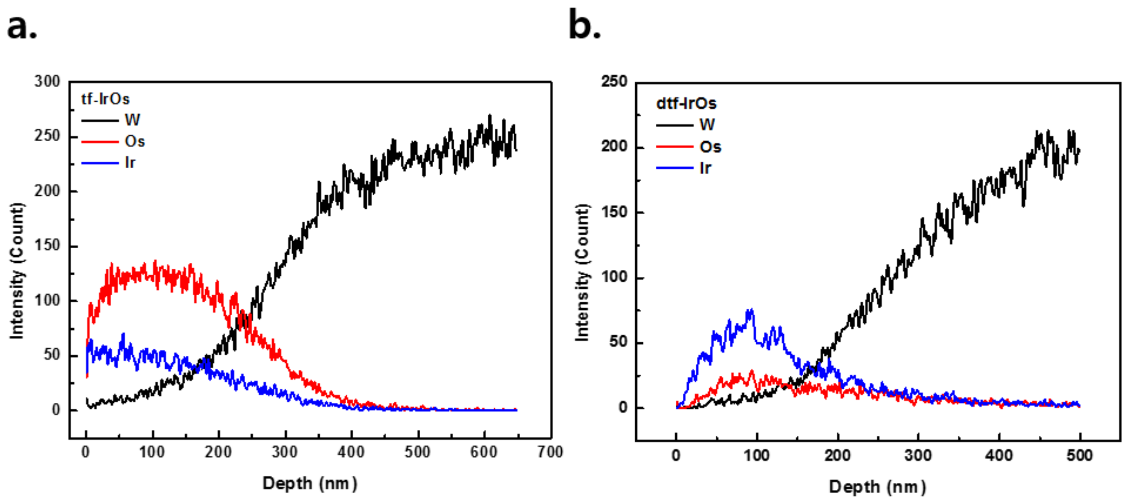

Supplementary Figure 4: SIMS depth profile for (a) tf-$Ir_{25}Os_{75}$ and (b) dtf-$Ir_{25}Os_{75}$.

Supplementary Note 4

Depth profile of $Ir_{25}Os_{75}$: The film thickness was identified with secondary ion mass spectroscopy (SIMS, ION-TOF, TOF.SIMS 5) with 10 kV accelerating Cs^+ ions. For this measurement, $Ir_{25}Os_{75}$ thin film layers were deposited on polycrystalline tungsten substrates to clearly identify the species that are in the thin film. The thickness of the as-prepared sample was found to be about 400 nm, while that of the dealloyed sample was about 250 nm. While the whole thin film composition is close to 71% Os before dealloying and between 25-30% Os after dealloying, the very near-surface region (15-20 nm) of the dealloyed film has less than 0.05% Os. Therefore, the contribution of Os-induced electronic effects to the electrochemical activity of dtf-$Ir_{25}Os_{75}$ should be negligible.

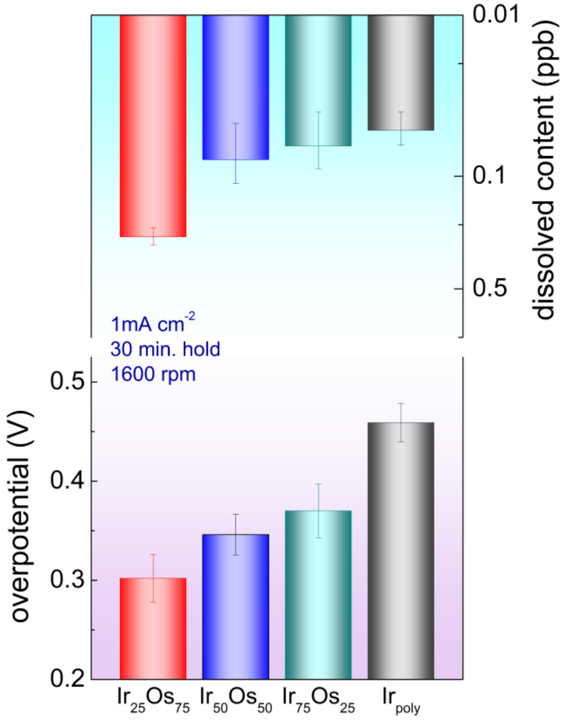

Supplementary Figure 5: Activity-Stability trends during OER for a series of dealloyed $Ir_xOs_{(1-x)}$ alloys. Error bars are the standard deviation after 5 experiments.

Supplementary Note 5

Activity-stability trends for varying alloy composition: Activity for OER and stability of oxide materials for a series of dealloyed thin film $Ir_xOs_{(1-x)}$ materials shows that activity is inversely proportional to stability. Changes in porosity due to the dealloying process from each $Ir_xOs_{(1-x)}$ composition indicate the best balance in activity and stability is found for dtf-$Ir_{25}Os_{75}$.

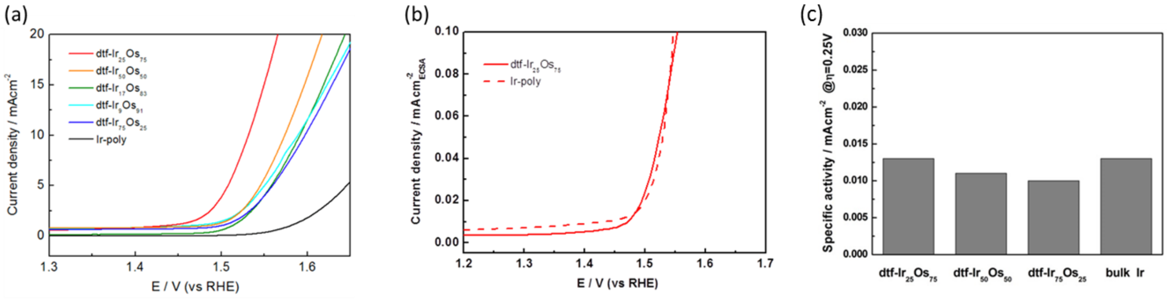

Supplementary Figure 6: (a) OER polarization curves for dtf-$Ir_{75}Os_{25}$, dtf-$Ir_{50}Os_{50}$, dtf-$Ir_{25}Os_{75}$, dtf-$Ir_{17}Os_{83}$, and dtf-$Ir_{9}Os_{91}$. (b) ECSA normalized OER polarization curves for dtf-$Ir_{25}Os_{75}$ and Ir-poly. (c) Specific OER activity for all dtf-$Ir_{x}Os_{(1-x)}$ and Ir poly materials.

Supplementary Note 6

Activity variation with alloy composition: OER activity changes with alloy composition. As shown in Figure S6 (a), the order of activity was dtf-$Ir_{25}Os_{75}$ >> dtf-$Ir_{50}Os_{50}$ > dtf-$Ir_{17}Os_{83}$ ≈ dtf-$Ir_{9}Os_{91}$ ≈ dtf-$Ir_{75}Os_{25}$ > Ir-poly, which is consistent with the morphology and ECSA as shown in Supplementary Figure 2. The normalization of OER polarization curves by ECSA and corresponding specific activity measured at 0.25V overpotential (b and c) shows that the all dtf-$Ir_{x}Os_{1-x}$ and Ir poly materials have similar intrinsic OER activity, demonstrating that the activity enhancement is more likely due to an increase in surface area rather than a consequence of the presence of Os. These data demonstrate that the dtf-$Ir_{25}Os_{75}$ has the optimal alloy composition for developing a highly active, nanoporous morphology. Possible explanations for the lower performance of the other alloys include limited evolution of porosity due to Ir passivation at lower Os contents or porosity collapse due to insufficient Ir connectivity at higher Os contents, which in both cases result in a decrease in ECSA and activity.

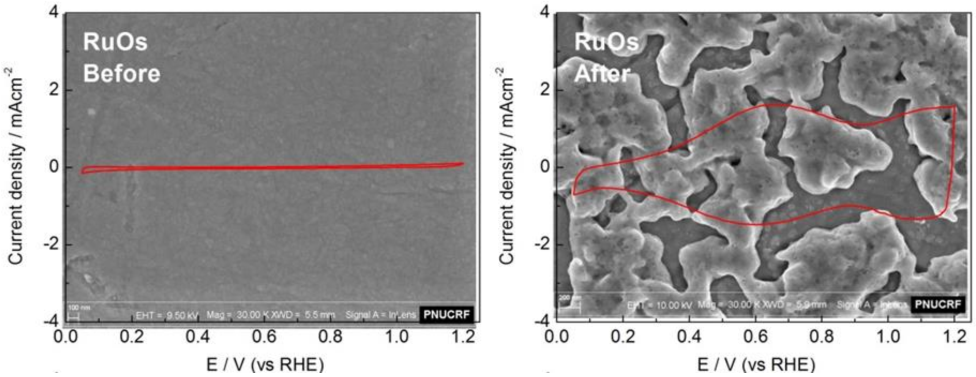

Supplementary Figure 7: Scanning electron micrograph (SEM) and CVs for $Ru_{25}Os_{75}$ before and after dealloying. Images are 4 x 3 μm^2.

Supplementary Note 7

Morphology of dealloyed RuOs materials: Unlike observed for dtf-$Ir_xOs_{(1-x)}$ materials, dealloying of $Ru_xOs_{(1-x)}$ leads to a much wider porous structure with larger pore sizes that are in line with simultaneous dissolution of Os and higher Ru dissolution rates relative to Ir. Even though the final surface area of dtf-$Ru_xOs_{(1-x)}$ is similar to dtf-$Ir_xOs_{(1-x)}$, the higher dissolution rate of Ru negates the gain in activity relative to the IrOs alloy system.

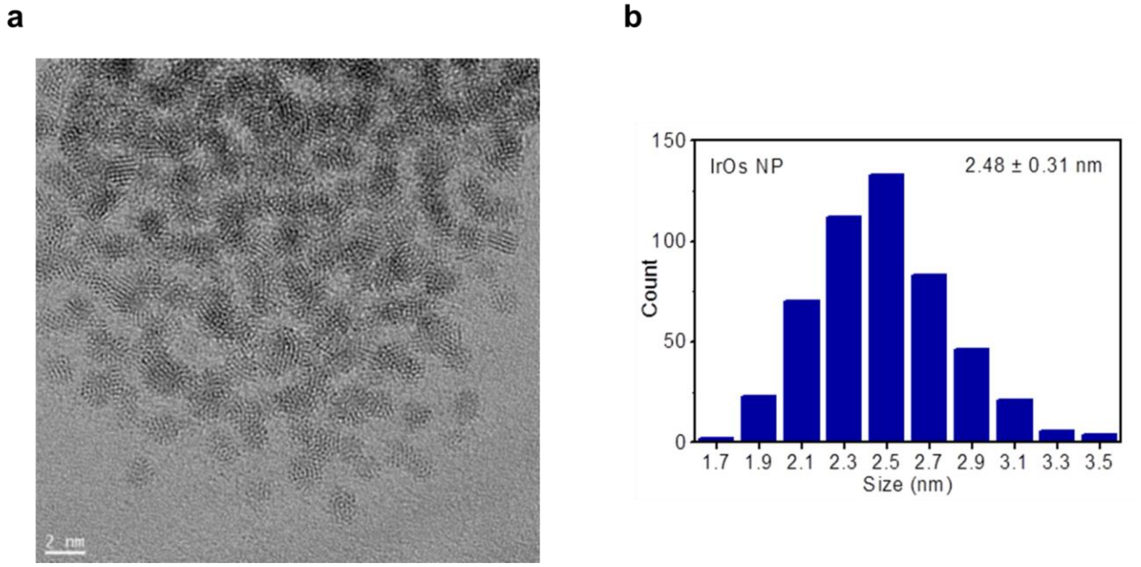

Supplementary Figure 8: TEM image **(a)** Particle size distribution **(b)** for $Ir_{50}Os_{50}$.

Supplementary Note 8

Size distribution histogram of IrOs nanoparticles: Particle size distribution for $Ir_{50}Os_{50}$ was obtained by TEM imaging (as shown in a) and considering 500 random nanoparticles. The average size is 2.48 nm ± 0.31 nm as the standard deviation. We note that the synthesis of $Ir_{25}Os_{75}$ nanoparticles was not possible due intrinsic difficulties with the synthesis method at increased Os content.

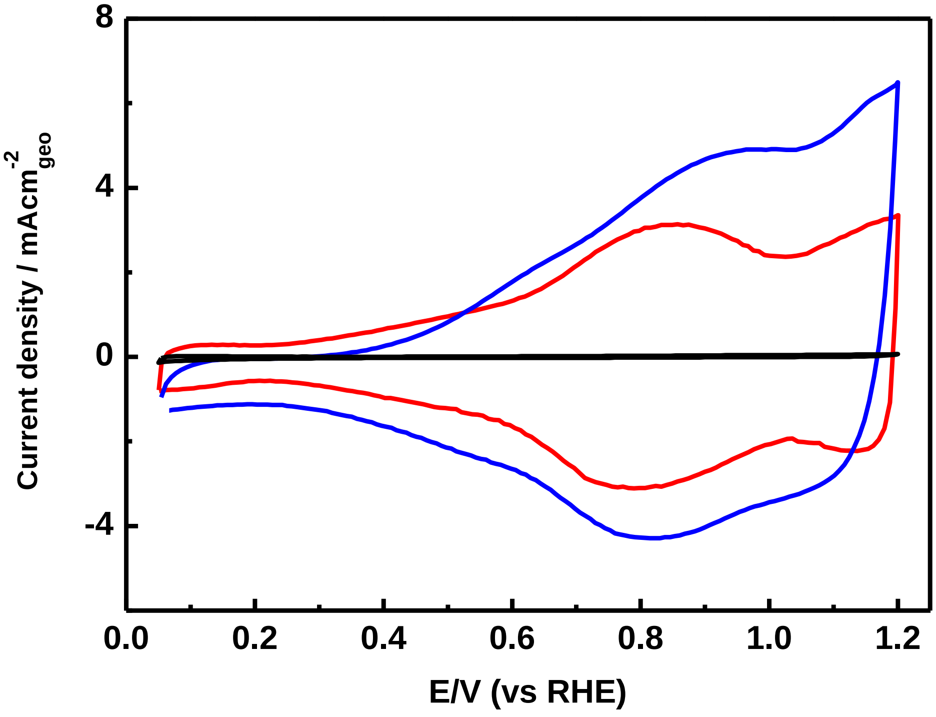

Supplementary Figure 9: Cyclic voltammetry for Ir-poly (black), dtf-$Ir_{25}Os_{75}$ (red), dnp-$Ir_{50}Os_{50}$ (blue).

Supplementary Note 9

ECSA comparison: The electrochemical double-layer capacitance in the CV curves shows that the ECSA is higher for dnp-$Ir_{50}Os_{50}$ than for dtf-$Ir_{25}Os_{75}$. The electronic properties of Ir seem to be similar for both systems regardless of different morphology and ECSA, as the positions of OH_{ad} peaks are almost the same.

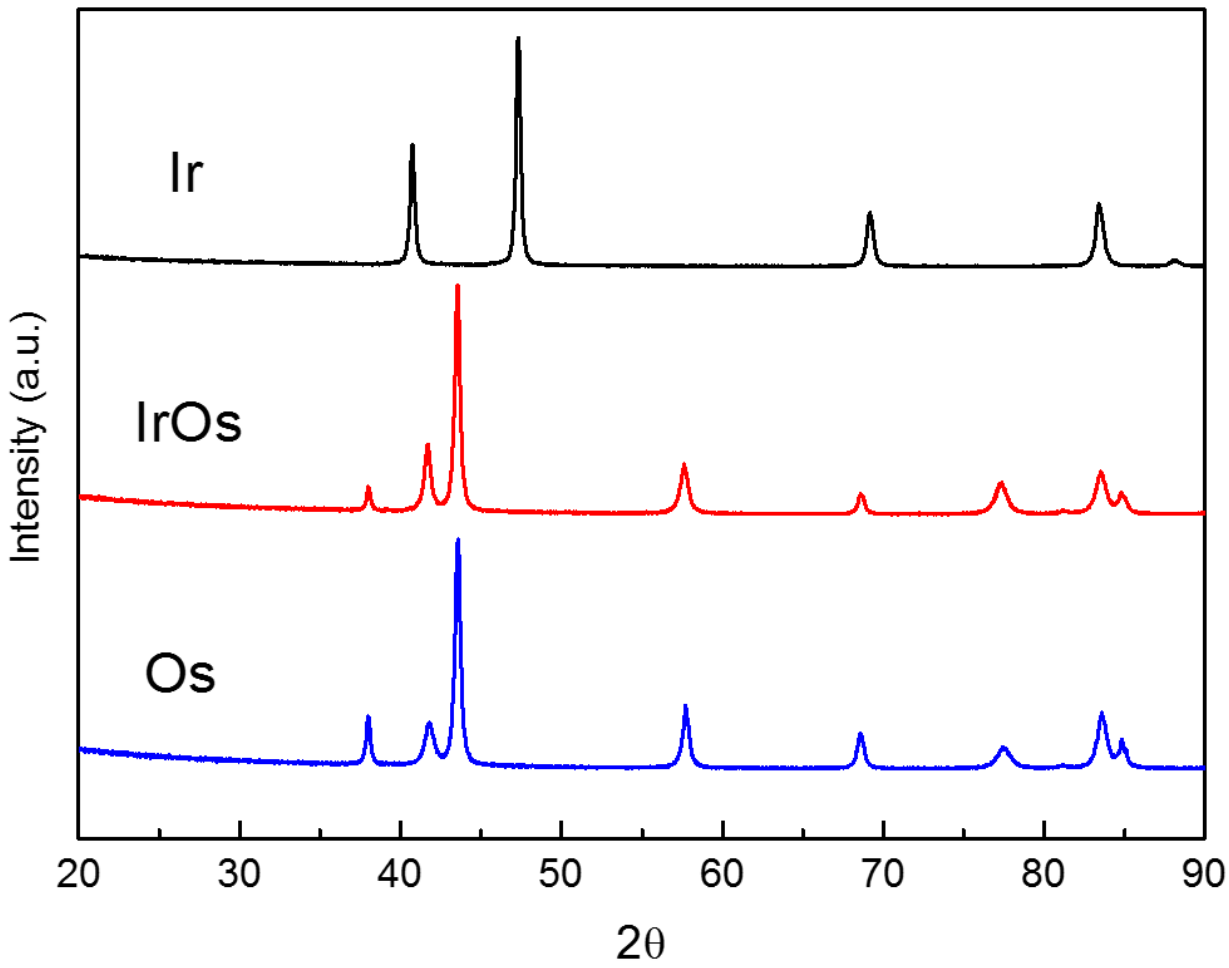

Supplementary Figure 10: XRD spectra for Ir-poly, Os-poly, and IrOs alloy

Supplementary Note 10

Crystalline structure of $Ir_{25}Os_{75}$: It was confirmed from XRD patterns that the prepared $Ir_{25}Os_{75}$ alloy thin film was in a single phase solid solution of hcp structure of Os rather than fcc structure of Ir without any side peaks, indicating that Ir atoms were homogeneously distributed in the Os lattice.

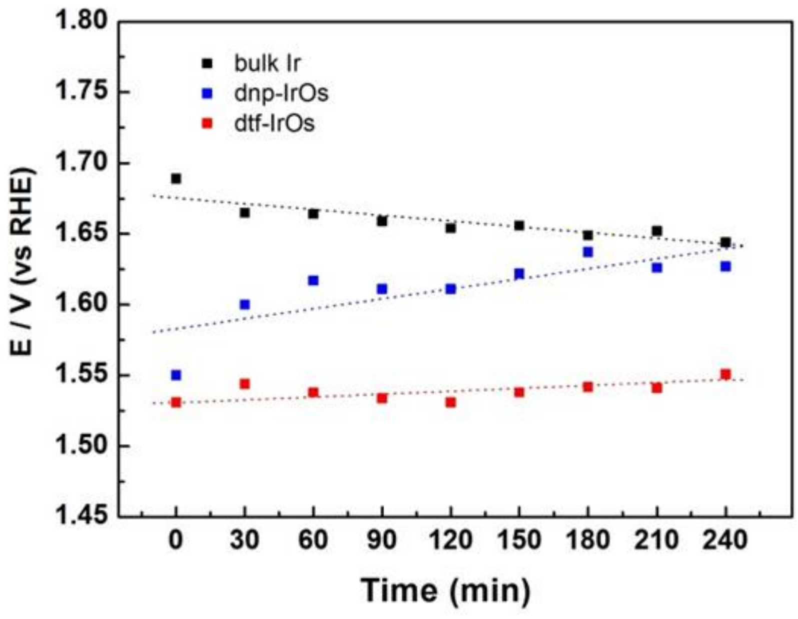

Supplementary Figure 11: Extend steady state OER polarization for Ir-poly, dtf-IrOs and dnp-IrOs catalysts under constant current 1 $mAcm^{-2}$.

Supplementary Note 11

Extended polarization measurements: Constant current polarization curves demonstrate the steady state activity profile, corroborating the cyclic voltammetry activity-stability evaluation. Also it shows that the catalysts can still preserve the high activity over extended periods of time, but knowingly that the continuous dissolution rates they will eventually loose activity.

Supplementary Table 1: Conductivity, Mobility, and carrier density for dtf-IrOs and dnp-IrOs obtained by the 4-probe van der Pauw method.

	**Conductivity [S cm^{-1}]**	**Mobility [cm^2 V^{-1} s^{-1}]**	**Carrier density [cm^{-3}]**
**dtf-$Ir_{25}Os_{75}$**	1.60×10^{-1}	8.99×10^{-2}	1.11×10^{19}
**dnp-$Ir_{50}Os_{50}$**	3.17×10^{-2}	1.57×10^{-2}	1.26×10^{19}

Supplementary References

1. Mccrory, C. C. L., Jung, S., Peters, J. C. & Jaramillo, T. F. Benchmarking Heterogeneous Electrocatalysts for the Oxygen Evolution Reaction. *J. Am. Chem. Soc.* **135,** 16977–16987 (2013).
